# Valorization of *Opuntia ficus-Indica* Pads and Steel Industry FeCl_3_-Rich Rejection for Removing Surfactant and Phenol from Oil Refinery Wastewater Through Coagulation-Flocculation

**DOI:** 10.5696/2156-9614-10.28.201204

**Published:** 2020-12-07

**Authors:** Ouafae Dkhissi, Mohammed Chatoui, Ahmed El Hakmaoui, Meriem Abouri, Yassine Kadmi, Mohamed Akssira, Salah Souabi

**Affiliations:** 1 Laboratory of Physical Chemistry and Bioorganic Chemistry, Faculty of Sciences and Techniques Mohammedia, Hassan II University of Casablanca, Morocco; 2 Engineering Laboratory of Water and Environment, Faculty of Sciences and Techniques Mohammedia, Hassan II University of Casablanca, Morocco; 3 University of Lille, Lille, France

**Keywords:** valorization, FeCl_3_, coagulation flocculation, removal, phenol, surfactant

## Abstract

**Background.:**

Refinement of crude vegetable oil generates a large amount of wastewater and is a source of water pollution due to the presence of surfactants and phenols. Phenols are toxic aromatic compounds that can be lethal to fauna and flora, entraining the deceleration or blocking of the self-purification of biological treatments. In addition, surfactants can limit biological processes by inhibiting microorganisms that degrade organic matter.

**Objectives.:**

The aim of the present study was to evaluate the treatment of refinery rejects loaded with phenols and detergents by coagulation flocculation using cactus pads (*genus Opuntia*) as a bio-flocculant and 30% iron(III) chloride (FeCl_3_) for surfactant and phenol removal. In addition, operating costs were evaluated for these pollution mitigation methods.

**Methods.:**

The effectiveness of cactus pads as a bio-flocculant and 30% FeCl_3_ for surfactant and phenol removal were studied using a jar test. The study was conducted on vegetable oil refinery wastewater from a refinery company in Casablanca, Morocco.

**Results.:**

The pollution load in wastewater varied widely from day to day. We evaluated the effect of cactus juice and 30% FeCl_3_ on high and low pollution loads. *Opuntia* pads showed a favorable potential for the treatment of low pollution load wastewater, with 78% and 90% of surfactant and phenol removed, respectively. However, the removal of high pollution load was less effective (42% and 41% removal of surfactant and phenol, respectively). The turbidity of low and high pollution load was reduced by 98.85% and 86%, respectively. The results demonstrate that 30% FeCl_3_ can effectively treat both low and high pollution loads (90% and 89% phenol removal, respectively, and 90% and 70% surfactant removal, respectively (optimal concentration 1.48 g/l). The turbidity was reduced by over 96% for both high and low pollutants.

**Conclusions.:**

The results of the present study indicate that cactus as a natural flocculant and reject rich in FeCl_3_ could be effectively used for the low-cost effective treatment of crude vegetable oil refinery rejects.

**Competing Interests.:**

The authors declare no competing financial interests

## Introduction

The refinement of crude vegetable oils is a source of water pollution, as the process generates large amounts of wastewater.^1^ Heavy metals, hydrocarbons, phenols, surfactants, pesticides, herbicides, and toxic sludge are some of the most common environmental pollutants requiring immediate abatement.^2–4^

Phenols are toxic aromatic compounds that can be lethal to fauna and flora, entraining the deceleration or the blocking of the self-purification of biological treatments. In addition, surfactants can limit biological processes by inhibiting microorganisms that degrade organic matter. Therefore, phenols and phenolic compounds should be removed from wastewater or decomposed before the water is discharged into the environment.^5^

A major challenge globally is the increasing demand for clean/safe water. Several strategies are used for water treatment, including coagulation-flocculation, adsorption by activated carbon, use of zeolites, membrane filtration, reverse osmosis, chemical precipitation, ion-exchange, electrochemical treatment, solvent extraction, and flotation for the removal of inorganic pollutants.^6^ Disadvantages of these technologies include generation of toxic sludge, high costs, limitations in the removal of low concentration pollutants, process complexity, membrane fouling, high chemical consumption, and high maintenance and operation costs.^7^

The use of natural coagulants for coagulation/flocculation treatment of wastewater has many advantages over chemical agents, including biodegradability, low toxicity, low residual sludge production, and low costs.^8^ Recently, natural organic polymers and polyelectrolytes have been used as flocculants and/or flocculation aids in river water and wastewater treatment.^9^

Cactus is highly flavored, has excellent nutritional properties, is globally distributed, is an important nutrient and food source, and has been used in medicine.^10^ *Opuntia ficus-indica* has excellent coagulation–flocculation and biosorption properties and has been extensively investigated for river water and wastewater decontamination.^11^ Other cactus species have also been successfully used as natural coagulants, largely attributed to the plant's mucilage, which has great water retention capacity due to its viscosity and storage of complex carbohydrate in the inner and outer pads.^12^

The *Opuntia ficus-indica* cactus is native to South America, but it is also found in arid and semi-arid regions. The family *Cactaceae* is known for its low-cost accessibility and production of mucilage, which is a complex carbohydrate and source of dietary fiber. A mixture of acidic and neutral polysaccharides, consisting primarily of arabinose, galactose, galacturonic acid, rhamnose and xylose, mainly constitutes the cactus cladodes.^13^

Natural coagulants are mainly comprised of carbohydrates (polysaccharides) and proteins.^14^ The principal advantages of the use of natural coagulants include organic and inorganic turbidity removal, reduction of true and apparent color, production of easy to deal with sludge, destruction of pathogens, algae and planktons, as well as the elimination of substances imparting odor and flavor. Natural coagulant usage is profitable since the treatment is low-cost, provides a steady pH level in the treated water and they are highly biodegradable.^8^

**Abbreviations**
*BOD5*: 5-day biochemical oxygen demand
*COD*: Chemical oxygen demand
*NTU*: Nephelometric turbidity units
*SIWW*: Steel industry wastewater
*UV*: Ultraviolet

The present work evaluates the use of cactus as a bio-coagulant for removing surfactant and phenol from oil refining wastewater via the jar test. This study also investigates the ability of rejected steel industry wastewater rich in 30% iron(III) chloride (FeCl_3_) generated by the Maghreb Steel Company to function as a coagulant for the removal of phenol and surfactant.^15^

## Methods

**Figure 1:**
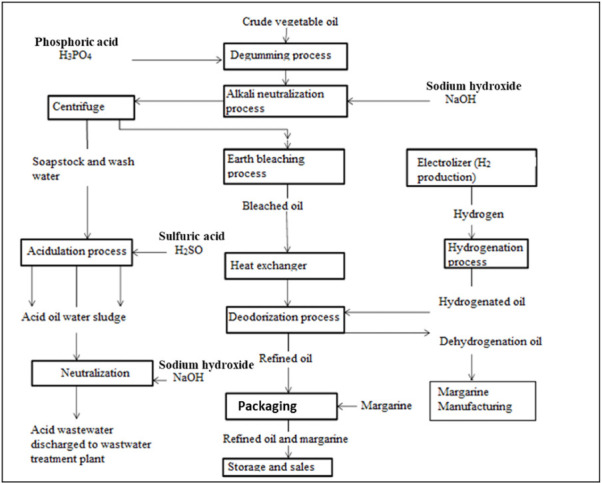
Simplified schematic diagram of vegetable oil refining processes: source of vegetable oil refinery wastewater (acid and processed wastewater)^2^

Industrial wastewater samples for this study were collected from the leading agro-industrial company in the area. In Africa, oil refining is one of the main agro-industrial activities. The Maghreb Steel Company has two processing units in Morocco, located in the region of Casablanca. The company performs oilseed milling and oil refining and produces olive oils and other edible oils, as well as various soaps. On average, the oil refinery generates 1200 m^3^/day of wastewater, which includes acid wastewater (80–270 m^3^/day) and process wastewater (570–1000 m^3^/day). The wastewater constitutes a stream originating from all the factory's process installations and equipment. Figure 1 presents a diagram of vegetable oil refining processes and the source of acid and process wastewater.^2^

### Wastewater origin

Wastewater was collected from an industrial wastewater treatment plant. The Maghreb Steel Company operates three segments: oilseed milling, oil refining, and soap making. The samples used for the present study consisted of process wastewater, which is a mixture of soap and glycerin wastewater. This mixture is one of the influents that enters the wastewater treatment plant.

### Analysis of physico-chemical parameters

Turbidity was measured using a turbidity meter (model 2100N, Hach). Chemical oxygen demand (COD) was determined using the open reflux method (5220-B). In this method, certain organic matters are oxidized by potassium dichromate (K_2_Cr_2_O_7_) in an acid medium. After digestion for two hours, the remaining unreduced K_2_Cr_2_O_7_ was titrated with ferrous ammonium sulphate to determine the amount of K_2_Cr_2_O_7_ consumed, after which the amount of oxidizable matter was calculated in terms of oxygen equivalents. Excess K_2_Cr_2_O_7_ was measured by Mohr's salt (sulfate of iron(II) and ammonium). The pH was determined using a pH meter (model 6209). Electrical conductivity was measured using a conductivity meter (Intelligent pH Meter YK-2001PH). Phenolic compounds were measured using Folin-Ciocalteu reagent, which develops a blue color that can be measured at 725 nm after incubating for one hour in the dark. Determination of surfactant was based on the formation of a soluble complex between the surfactant, which is an anionic compound, and methyl violet, which is a cationic compound, in toluene. Absorbance was measured at 615 nm. Dissolved oxygen was measured with a dissolved oxygen probe (HI 9143, Hanna Instruments). The 5-day biochemical oxygen demand (BOD_5_) is a method of analyzing organic matter in wastewater by biological means. The technique consists of bringing the wastewater rich in easily biodegradable organic matter into contact with a biomass which degrades the organic matter for a five-day period necessary for the complete degradation of organic matter.^16^

### Steel industry wastewater

Steel industry wastewater (SIWW) is rich in FeCl_3_, and has previously been used as a coagulant for the treatment of wastewater charged in dyes produced by the Maghreb Steel Company in Morocco.^15^ The cactus bio-coagulant characteristics are presented in Table 1. Steel industry wastewater results from the elimination of the oxide coating by a chemical scouring with hydrochloric acid (HCl) according to the reactions below:

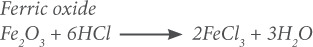


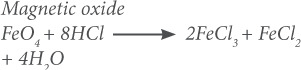


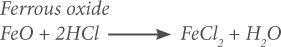



**Table 1:** Characteristics of Bio-Coagulant (Cactus Juice)

**Parameter**	**Value**
Appearance	Viscous liquid
Color	Green
pH	6.5
Density	1.008 Kg/l
Molecular weight (g/mol)	2.3–3.1
Contains about water	96%

The volume of released SIWW rich in FeCl_3_ at 30% produced by the Maghreb Steel Company greatly exceeded 1000 L/d. The rejection rich in FeCl_3_ can be recovered to be used for the treatment of refinery wastewater by coagulation flocculation on a pilot scale to examine feasibility of the treatment.

### Jar test: experimental conditions

A laboratory-scale evaluation of chemical coagulation and flocculation was performed using a six-place jar test apparatus. The experimental process consisted of three subsequent stages: an initial rapid mixing at 160 rpm for 10 minutes, followed by a slow mixing for 20 minutes at 30 rpm, and then a final settling step for 1 hour. Coagulation–flocculation was conducted with the optimized operational variables (COD, BOD_5_, TSS, oil and grease, temperature, turbidity, phenol, surfactant, pH, color, conductivity, total phosphorus, and nitrates) determined earlier. Six polyethylene beakers of equal volume were used to examine the different dosages of coagulant and initial pH in each run. The sample bottles were thoroughly shaken to resuspend any settled solids and the appropriate volume of sample was transferred to the corresponding jar test beakers.

After 60-minutes settling, the supernatant was withdrawn for analyses. To assess the efficacy of cactus on wastewater treatment, the following characteristics were determined: turbidity, COD and color.

The removal efficiency was calculated using Equation 1:

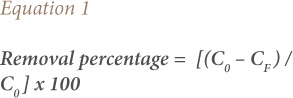
where, C_0_ and C_F_ are the initial and final values respectively of the studied parameter.

### Natural flocculant: preparation of *Opuntia ficus-indica* juice for use in wastewater remediation

*Opuntia ficus-indica* pads are a natural product, abundant in nature. They are biodegradable and do not threaten the environment. The *Opuntia* used in the present study was from Mohammedia in the west of Morocco, and the cladodes were transported immediately to the laboratory to be used for juice extraction. They were repeatedly washed with water to remove dirt particles and dissections of fresh *Opuntia* pads were performed by hand. First, the skin was peeled from the pad, cut into small pieces and mixed using a domestic mixer, then the juice was filtrated in a 500-micron diameter filter. The bio-flocculant was stored in a glass bottle in a refrigerator at 4°C until use. The bio-flocculant is relatively stable and can maintain its coagulant capacity for several days outside any conservation system. The bio-flocculant was diluted to 10% for use in the present study.

**Figure 2:**
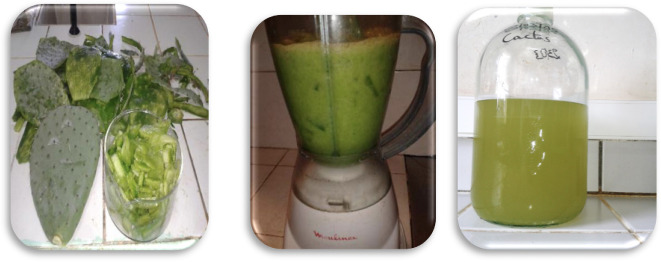
Photos of pads and cactus juice (Opuntia ficus-indica)

The bio-coagulant was a viscous liquid of green color, with a pH of 6.5, and miscible with water *(Figure 2)*. The characteristics of the bio-coagulant are shown in Table 2.

**Table 2:** Characteristics of Steel Industry Wastewater Rich in FeCl_3_

**Parameter**	**Value**
pH	<1
Conductivity (us/cm)	20
Fe^3+^ (g/l)	101.3
FeCl_3_ (g/l)	295

Abbreviation: Fe^3+^, iron(III).

## Results

**Figure 3:**
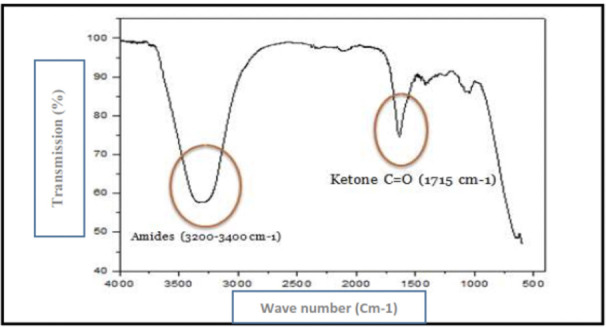
Infrared adsorption spectrophotometer of cactus juice

The infrared absorption spectrophotometer of cactus juice is shown in Figure 3.

**Figure 4:**
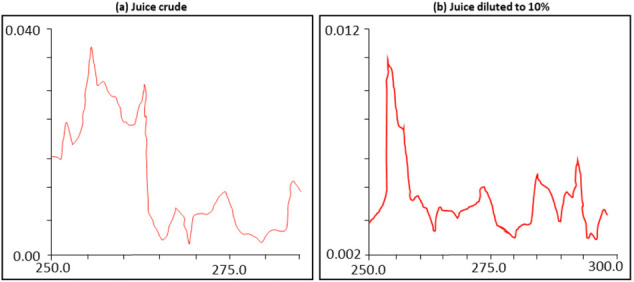
Ultraviolet absorption spectra of cactus juice

In addition, the ultraviolet (UV) absorption spectra of cactus juice are presented in Figure 4.

Figures 3 and 4 show the results of infrared absorption spectrophotometry and UV absorption spectra of cactus juice. The results of the infrared spectrum make it possible to detect the presence of wide and superimposed bands in the 3200–3500 cm^−1^ region which are due to the presence of amides. In addition, the presence of an adsorption peak of 1715 cm^−1^ was due to the presence of C = O ketone. In addition, the results of the analyses carried out by the UV spectroscopy show the presence of an absorption wavelength around 255 nm.

### Wastewater characteristics

Table 3 shows the mean values of the physicochemical characteristics measured in the processed wastewater from the industrial production of vegetable oil.

**Table 3:** Characteristics of Process Wastewater in a Vegetable Oil Industry Wastewater Treatment Plant

**Parameters**	**Process wastewater**		

	**Mean**	**Minimum**	**Maximum**
pH	10.01	9.45	10.85
Conductivity (ms cm^−1^)	32.5	21.7	42.3
Temperature (°C)	34	23	36
TSS (mgL^−1^)	7415	5630	10170
COD (mg L^−1^)	48241	22400	53563
BOD_5_ (mg L^−1^)	15856	7889	20179
Oil and grease (mg L^−1^)	5495	504	6542
Phenol (mg L^−1^)	69.5	45.69	85.49
Total phosphorus (mg L^−1^)	335	84.88	586
Surfactant (mg L^−l^)	80.33	31.21	121
DO (mgO_2_ L^−1^)	1.28	0.13	2.28
Turbidity (NTU)	3251	1119	3922
BOD_5_/COD	0.32	0.35	0.37

Abbreviations: TSS, total suspended solids; COD, chemical oxygen demand; BOD_5_, 5-day biochemical oxygen demand; mgO_2_, magnesium peroxide; NTU, nephelometric turbidity units; DO, dissolved oxygen.

**Figure 5:**
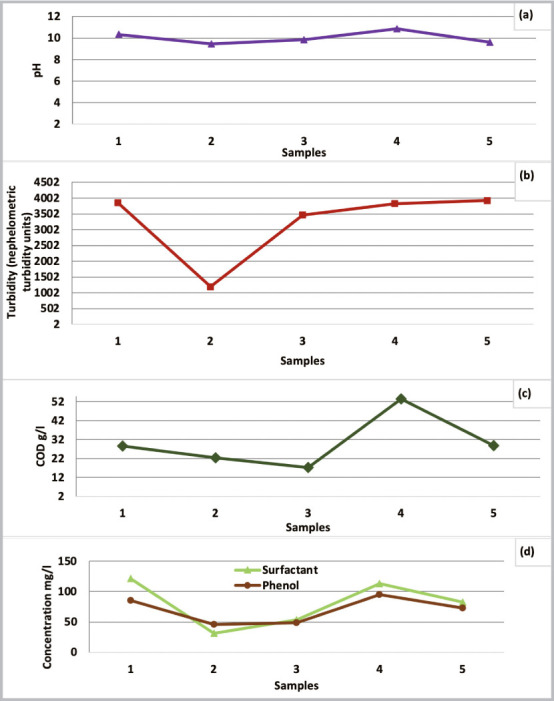
Variation of (a) pH; (b) turbidity (nephelometric turbidity units); (c) COD (mg/l); and (d) surfactant and phenol of process wastewater

The characteristics of the wastewater samples shown in Table 3 are based on five measurements taken once per week over five weeks. The variation of physicochemical parameter values (pH, turbidity, COD, surfactant, and phenol) are shown in Figure 5.

As shown in Figure 5 (a), the pH of the process effluent was basic and did not significantly change over five weeks.

Turbidity fluctuated for all sampling periods *(Figure 5 (b))*; the maximum turbidity value was approximately 4000 nephelometric turbidity units (NTU). This value decreased to approximately 1000 NTU during the second week then increased to reach approximately 3400 NTU, which was due to the variation of the physicochemical quality during the sampling period. Industrial effluents often exhibit physicochemical characteristics over time, which is due to the production process as shown by Chatoui *et al*.^17^

The effluent was characterized by a high organic load in terms of COD and BOD5, with an average of 48,241 mg/l and 15,856 mg/l, respectively. Figure 5 (c) shows the weekly variation of COD. In the three first weeks, COD values were low, but in the fourth week COD increased to approximately 53,563 mg/l, and then returned to the initial value in the fifth week.

Wastewater BOD_5_/COD ratios were calculated to evaluate the potential biodegradability of the organic compounds in the effluent. A BOD_5_/COD ratio > 0.3 suggests that organic compounds present in the effluent are biodegradable.^18^ However, the relative effluent biodegradability fluctuated weekly.

A high concentration of surfactant and phenol were measured. An average of 80.33 mg/l and 69.5 mg/l for surfactant and phenol were recorded, respectively *(Table 3 and Figure 5 (d))*. Concentrations of surfactant and phenol were high in the first week then decreased. In the third week, the concentration of these two parameters increased, reached maximum concentration in the fourth week (113 and 95 mg/l of surfactant and phenol, respectively), and then decreased again in the fifth week.

**Figure 6:**
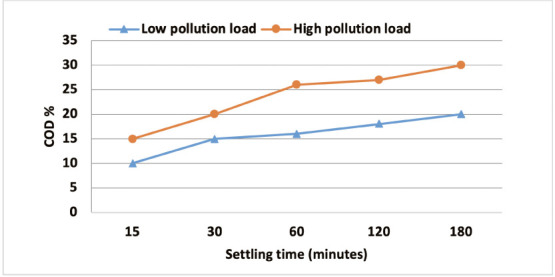
Effect of decantation on the reduction of the pollution load

**Figure 7—:**
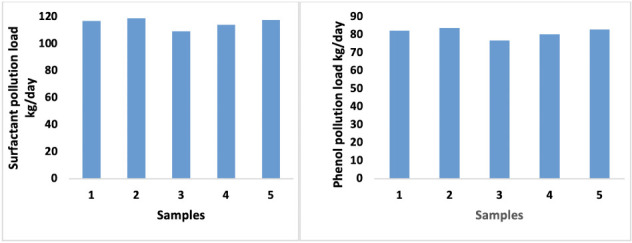
Variation of pollution load of phenol and surfactant in process wastewater

We performed a comparative study of the effect of cactus on the elimination of surfactant and phenol from high and low pollution loads. The effect of *Opuntia* juice on the removal of COD, surfactant and phenol are shown in Figures 6 and 7, respectively.

The natural decantation of the releases studied as a function of decreasing pollution load (COD) is shown in Figure 6. The removal of COD increased as the settling time increased, which was due to the settling behavior of suspended particles and other floc particles that are collected during their movement in the solution to form settled particles.^19^

As shown in Figure 7, the pollution load of vegetable oil refinery wastewater, in terms of phenol and surfactant, can reach nearly 120 kg/day of surfactant and 83 kg/day of phenol.

Phenol is highly toxic at high concentrations and may be absorbed by the skin. Treatment of water containing phenol is of high concern because of the high toxicity of phenolic compounds.^20^

## Discussion

**Figure 8:**
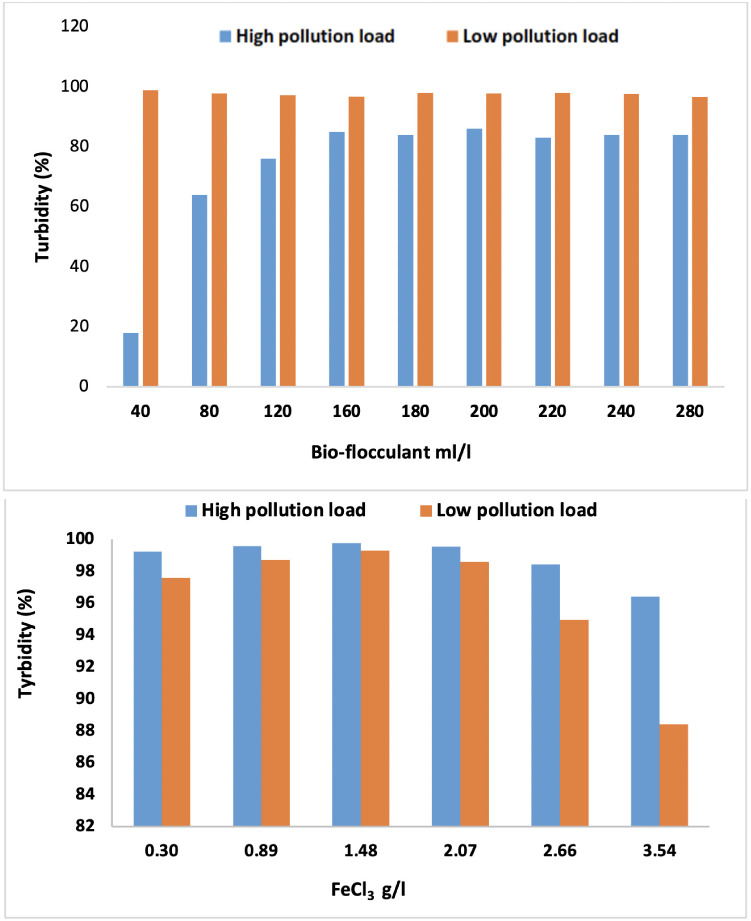
Effect of Opuntia juice and FeCl_3_/SIWW on turbidity removal with high and low pollution load

The removal efficiency of turbidity obtained by coagulation-flocculation using the *Opuntia* and FeCl_3_ (30%)/SIWW are respectively illustrated in Figure 8.

The experiments were performed with different volumes of cactus, ranging between 40 ml/l and 280 ml/l and different concentrations of SIWW, ranging between 0.3 and 3.54 g/l. As can be seen in Figure 8, the turbidity removal increased as the cactus volumes increased from 40 ml/l to 160 ml/l with high pollution load and achieved 85% turbidity removal. Increasing cactus volume greater than 160 ml/l resulted in a stable removal of 84%. At a low pollution load, the turbidity removal was great at 40 ml/l with 98.85%. It was observed that even though the volumes were substantially increased, the turbidity removal was relatively unaffected. Many studies investigated the removal of turbidity using cactus and showed more than 98% of flocculating activity.^21^ A similar study conducted by Bouatay *et al.* using cactus mucilage combined with aluminum sulphate showed that the turbidity removal was 91.66%.^22^ This was compared with the finding of a study conducted by Yin *et al.* using powder cactus which reported 98.2% turbidity removal.^23^ *Opuntia* juice has an excellent effect on the turbidity removal from low and high pollution loads, but in the low load case the removal was greatest.

The SIWW had the best performance in removing turbidity of vegetable oil industry wastewater, achieving greater than 99% efficiencies for low and high pollution loads with an optimal dose of 1.48 g/l. Use of SIWW, however, was not statistically different from the use of ferric chloride as a coagulant for the reduction of turbidity from three rejections generated at different points in the city of Salé, where the removal achieved was 96%.^24^ Another study by Feria-Diaz *et al.* on the treatment of raw water samples from the Sinú River by natural coagulants achieved an elimination of turbidity up to 95% using *Moringa oleifera* seed.^8^ Similar results were reported by Muthuraman and Sasikala, using natural coagulants for the removal of turbidity from drinking water.^25^ *Moringa oleifera*, *Strychnos potatorum* and *Proteus vulgaris* reduced turbidity to 5, 10, 1 NTU, respectively, from 500 NTU. Another study conducted by Sanchez-Martin *et al* demonstrated that the use of two purified coagulants extracted from *Moringa oleifera* had significantly better turbidity removal efficiency for the coagulation of surface water from the Meuse river in the Netherlands.^26^

### Surfactant removal

**Figure 9:**
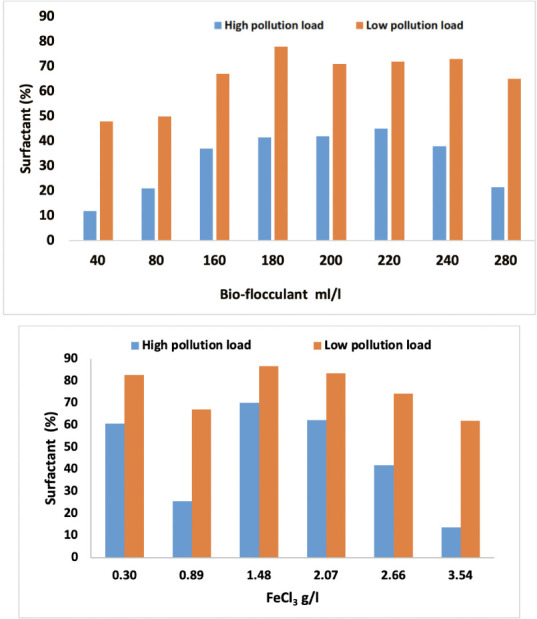
Effect of Opuntia juice and FeCl_3_ 30% on surfactant removal in high and low pollution loads

The surfactant removal efficiencies obtained by coagulation-flocculation using the *Opuntia* juice and FeCl_3_ (30%) are illustrated in Figure 9.

The toxicity of surfactant is related to the hydrophobicity of the molecule and the length of the alkyl chain, and it can be reduced when the number of ethylene oxide units increases, decreasing hydrophobicity.^27,28^

The process wastewater generated from vegetable oil refinery has high surfactant concentrations.^29^ It was observed that the removal of the surfactants increased by increasing the volume of cactus juice until the volume reached 180 ml/l, and then it decreased, for both high and low pollution loads. The above trend may be attributed to the fact that the colloidal organics further destabilized over and above the optimum dosage. The greatest removals of surfactant in high and low pollution loads were 78% and 45%, respectively. The cactus volume had a significant effect on the removal efficiency of surfactant. It had a better elimination value of surfactant from the low pollution load than the high pollution load, which contained high amounts of polar and nonpolar organic constituents, expressed in term of COD. Excessive use of any type of surfactant and disposal has the potential to seriously affect ecosystems. Moreover, in the case of SIWW, the optimum removal of surfactant was achieved at 1.48 g/l, reaching 86.8% and 70.1%, with low and high pollution loads, respectively. Moreover, it seems that the SIWW gave more favorable results than *Opuntia* juice for reducing surfactant from process wastewater.

Terzic *et al*. compared the efficiency of two processes for the elimination of the surfactant contained in municipal wastewater, the classical unit of mechanical/biological treatment and a biological engine with a membrane.^30^ The latter could attain maximum effectiveness. Another study demonstrated that adsorption had the greatest efficiency on the removal of cationic surfactant at 90%.^31^

### Phenol removal

**Figure 10:**
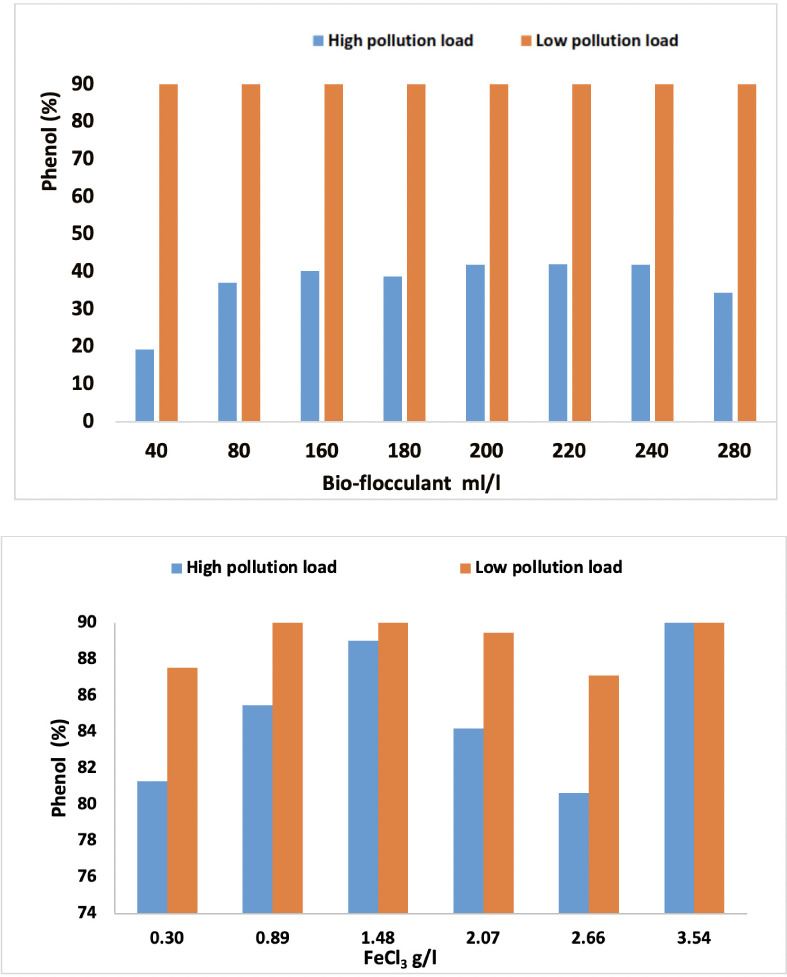
Effect of Opuntia and FeCl_3_ 30% on phenol removal in high and low pollution loads

Figure 10 shows the effects of *Opuntia* and SIWW on the removal of phenol from wastewater via the coagulation-flocculation process.

Phenol and phenolic compounds are among the most widespread chemical pollutants of industrial wastewater. Phenols exhibit a high degree of toxicity in the environment and for human health.^32,33^ Phenol degradation has been a topic of scientific interest for a number of decades.

Removal of phenol using various volumes of cactus ranged between 19.3% and 41.9% for high pollution loads and 90% for low pollution loads. Cactus juice treatment was more effective with high pollution loads, which were more complex mixtures containing dissolved organics, suspended solids, and heavy metals. Naghibi *et al.* studied phenol removal using *Raphanus sativus* root and its juice.^34^ The highest removal efficiency was 90%, which was similar to the highest removal efficiency (91.6%) obtained using local sawdust to reduce phenol concentration.^35^ Optimal phenol removal was achieved at 1.48 g/l of SIWW, with highest removals of 90% and 89% with low and high pollution loads, respectively. These results showed little difference in the elimination of phenol for low and high pollution loads of SIWW rich in FeCl_3_. In a comparative study using FeCl_3_, polyaluminum chloride, and chitosan to reduce phenol from aqueous solution, phenol removal was 45% with FeCl_3_, 36.4% with polyaluminum chloride, and 29.4% with chitosan.^36^ Similarly, comprehensive removal of phenol can be achieved by the peroxi-electrocoagulation method. In one study, 92% of phenol was removed at a current density of 0.10 A/dm^2^ and pH of 2.0 using mild steel as the anode and stainless steel as the cathode.^37^

**Figure 11:**
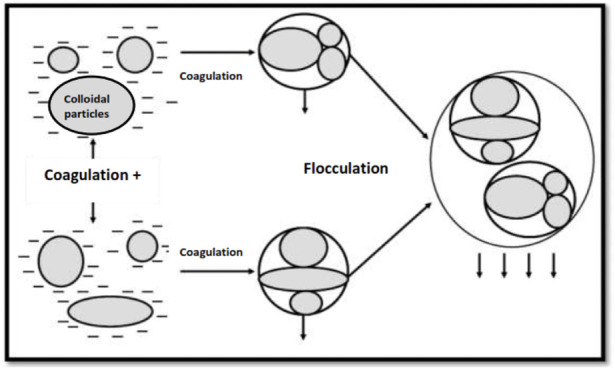
Coagulation-flocculation mechanism^38^

The process of coagulation-flocculation facilitates the removal of suspended solids and colloidal particles.^17^ This technique involves essentially neutralizing the electric charge (zeta potential tends towards 0) and thus promotes the bringing together of the particles with a view to their agglomeration and then settling, which results in well-treated water which can be recovered. Figure 11 illustrates the mechanism of elimination of phenol, detergents, or others.

## Conclusions

The present study evaluated the use of cactus as a bio-coagulant for removing surfactant and phenols from oil refining wastewater via the jar test. In addition, it investigated the ability of rejected steel industry wastewater rich in 30% FeCl_3_ generated by the Maghreb Steel Company to function as a coagulant for the removal of phenol and surfactant.

The results of the present study show that cactus juice has the capacity to remove surfactants and phenols from low and high pollution loads, with varying efficiencies. The cactus juice was effective in removing surfactant and phenol from low pollution loads (removal efficiency of 78% for surfactant and 96.8% for phenol). However, cactus juice was less effective for high pollution loads (45% removal of surfactant and 42% removal of phenol). This suggests that cactus may be useful for treating oil refinery wastewater with a low pollution load. In addition, SIWW rich in ferric chloride (30%) effectively removed surfactant (70% low pollution loads and 90 for high pollution loads) and phenol from low and high pollution loads, with efficiencies reaching 90% approximate in both conditions.

The natural cactus flocculant and industrial waste rich in FeCl_3_ significantly reduce the pollution of the wastewater chosen for the study, rich in phenol and surfactants.
